# Subjective Socioeconomic Status, Class Mobility and Health Disparities of Older People

**DOI:** 10.3390/ijerph192113955

**Published:** 2022-10-27

**Authors:** Xiaoxing Ran, Tiange Huang, Gong Chen

**Affiliations:** Institute of Population Research, Peking University, Beijing 100871, China

**Keywords:** subjective socioeconomic status, class mobility, health disparities, older people

## Abstract

The aim of this study is threefold: (1) investigate the impact of subjective socioeconomic status on the health of older people with a further exploration of its extent, (2) explore the relationship between diverse class mobility trajectories and health disparities among older people, and (3) examine the health disparities among older people with different subjective socioeconomic statuses in groups of various class mobility. The data for this study came from the Chinese General Social Survey (CGSS) of 2017, and regression and moderation models are used. The results indicate that for each level of increase in the subjective socioeconomic status of older people, the health score significantly increased by 0.159. The health status of older people who experienced upward class mobility is significantly better than those who did not; therefore, different class mobility trajectories also shape health disparities. The moderation role of class mobility on the impact of subjective socioeconomic status on the health of older people is also confirmed. The results provide further support for the subjective socioeconomic status having a significant impact on the health of older people with different class mobility trajectories. An upward class mobility trajectory somewhat diminishes this influence. Therefore, policy interventions should be provided to enhance the subjective socioeconomic status of older people and increase their opportunities for upward class mobility.

## 1. Introduction

Subjective socioeconomic status is defined as the self-appraisal of one’s location in a socioeconomic system [1]. Terms that are sometimes used synonymously are perceived social position [2] and subjective social status [3,4,5]. It is contrasted with objective socioeconomic status. Numerous studies have focused on objective indicators, such as education [6,7], occupation [8,9], and income [10]. Objective stratification is consistent with the overall structure of subjective identity [11]. The differences in people’s socioeconomic statuses lead to social stratification, which is a widespread phenomenon in real life, usually based on certain division criteria for members, with people of the same socioeconomic status being grouped into the same class [12]. Subjective socioeconomic status is a key indicator of social classification that changes according to the stage of life. The change in one’s economic and social status over time forms the trajectory of class mobility. That is, class mobility is a change in the relative position of the people in a social stratification system [13].

Health disparities refer to simple variations across groups or differences after accounting for extensive causes [14]. They have been defined in different ways and sometimes are substituted with health inequalities in Europe or health inequity when the issue of social injustice is involved [15]. A variety of populations are involved when health disparities are explored [2], such as children [16,17], residents or adults [9,18,19], and older people [20,21,22,23]. Health disparities arise from two dimensions: the first concerns people’s underlying characteristics, and the second concerns the stage of life experience [24]. From the latter dimension, we believe that subjective socioeconomic status—as a subjective experience—is relative to health disparities. Meanwhile, the health disparities among older people will be more pronounced due to their extensive experience and, thus, deserve more attention.

All countries show consistent disparities in mortality and morbidity between persons with better socioeconomic statuses and poorer residents, even for those at the top of the charts regarding measures of economic success and human development [25]. The positive relationship between subjective socioeconomic status and health disparities has been confirmed in multiple ways [26,27,28,29,30]. Many studies found that the higher the socioeconomic status, the better the subject’s health status [24]. The reason for this is that people of a higher socioeconomic status are less likely to suffer from injuries due to their good working and living conditions [31] and higher accessibility to healthcare services [32]. Other impact mechanisms include a greater exposure to toxins, carcinogens, and violence; fewer resources (parks, libraries, and supermarkets); health-damaging behaviors such as cigarette use, excessive alcohol consumption, and a lack of exercise; and psychological states such as anger and feeling low levels of control, autonomy, and trust, all of which occur more frequently in socially disadvantaged populations [33]. Few studies used Chinese data to support the robust association between socioeconomic status and a diverse range of health outcomes, and even fewer have used indicators of subjective socioeconomic status.

The relationship between social class and health has long attracted much attention [34,35,36,37], but class mobility has not been included. Mobility is seen as a major way to reduce social inequality [38]. The effect of socioeconomic status on health inequalities is cumulative [39], whereby people who are chronically advantaged (or disadvantaged) have better (or worse) health, and social mobility can somewhat attenuate this cumulative effect [40]. Meanwhile, the heterogeneity of older people is a result of the differentiation of their life course [41]. Do various class mobility trajectories shape health disparities? No clear answer has been given in the literature.

Subjective socioeconomic status is the current perception, and class mobility trajectories represent people’s experiences. To provide a comprehensive presentation of how health disparities are created with diverse subjective socioeconomic statuses and class mobility trajectories, we need to combine the two influencing factors. At present, few studies have examined the relationship between subjective socioeconomic status, class mobility, and health disparities. Meanwhile, the health of older people needs urgent attention as it is closely related to the achievement of active aging and the strategic goal of a “Healthy China”. China has become the country with the largest elderly population in the world and has a rapidly increasing rate of aging. In the process of promoting healthy aging strategies, it is of great theoretical and practical value to study health disparities among older people since it is not only a personal issue, but also a social one, and dealing with health stratification and narrowing the health gap between classes is of great significance to achieve social justice and harmonious development [42]. With our study, we aim to shed light on the influence of subjective socioeconomic status on the health of older people and the role of class mobility in the formation of health disparities. The results will increase the information on health disparities of older people in China, providing a direction for social policy interventions.

## 2. Materials and Methods

### 2.1. Data

Data used in this study come from the Chinese General Social Survey (CGSS), sponsored by the Survey and Data Center of Renmin University of China. It is the earliest national, comprehensive, and continuous academic survey project in China, which started in 2003 [43]. The target population of the sampling scheme is all urban and rural households in 31 provinces/autonomous regions/municipalities directly under control of the Central Government (excluding Hong Kong, Macao, and Taiwan) in mainland China.

We used the CGSS data of 2017, which were based on a sample design from 2010, with the 2009 national population serving as the population-sampling frame. A stratified, multi-stage probability proportional to size (PPS) method was adopted. A key feature of the sampling scheme is the division of the survey population into two strata: the mandatory stratum (residents of the municipal districts of the more developed mega-cities) and the sample stratum (all residents except for the mandatory stratum). For the definition of developed mega-cities, the survey focused on 36 cities directly under the central government, provincial capitals, and sub-provincial cities; selected the three aggregate indicators of GDP, the total number of teachers, and the amount of foreign direct investment (FDI) actually used; from the perspectives of economic level, education level, and openness of the city; and used factor analysis to determine the top five cities in the mandatory tier. The overall survey population in the sample selection stratum consists of urban and rural households outside the mandatory stratum. The sampling stratum was divided into district and county strata (including county-level cities and counties), and factor analysis was conducted in the district and county strata using three indicators—population density, the proportion of the non-agricultural population, and per capita gross regional product—to obtain the comprehensive factor scores of each district and county within the district and county strata; based on the ranking of the comprehensive factor scores, the district stratum was further divided into 19 strata and the county stratum was further divided into 31 strata, thereby creating the sampling stratum. The total subdivision is 50 layers.

The CGSS data were adopted in this research for two reasons: First, they are representative due to their scientifically stratified sampling. The design scheme takes full account of the needs of national and different geographical estimations and provides scientific and detailed stratification of the overall survey, which improves the estimation accuracy to a certain extent; in particular, the handling of issues such as urban–rural sample matching, the establishment of self-representation strata, and the weighting of sample data reflect the scientific, efficient, and operable characteristics of this scheme. The composition of the sample largely matches the actual situation in China and has become the most important source of data for the study of Chinese society, and is widely used in research, teaching, and governmental decision making. The data have also been used in several studies of older people [44,45,46,47,48], indicating a representative sample. In this study, we collected a sample of people aged 60 years and over. After eliminating missing data, 3782 samples were obtained. The basic characteristics of older people are shown in Table 1. Second, the data are highly applicable due to their large size and the wealth of information. There are three modules in the questionnaire, namely, the Core Part (A), Social Network and Network Society (C), and Household Questionnaire(D), with a total of 783 variables. In the Core Part (A), there are questions on subjective socioeconomic class identification and health that are available in this research. Therefore, these data provide extremely valuable information for our study.

### 2.2. Variables

Subjective socioeconomic status is one of the independent variables in this article. It was measured with the question ‘On balance, your socioeconomic status belongs to?’, wherein the five options include upper, upper-middle, middle, lower-middle, and lower. People with a higher self-perceived rating of their socioeconomic status enjoy better health [33]. The other independent variable is class mobility. A self-anchoring scale in the form of a 10-rung ladder was used to measure subjective social status. Participants were given a drawing of a ladder with the following instructions: In our society, some people are at the top of the social ladder, and some are at the bottom. This card (show card 4) of the ladder should be viewed from the top-down. The highest ‘10’ represents the top of the ladder and the lowest ‘1’ represents the bottom of the ladder. We use the questions ‘Which grade do you think you were on 10 years ago?’, ‘Which grade do you think your family was when you were 14?’, and ‘Which grade do you personally stand in society at the moment?’ to represent class mobility in different time periods: the first is from the age of 14 to the present; the second is from 10 years ago to the present. We divided class mobility into three kinds: upward, constant, and downward.

Health status is the dependent variable. Health outcomes can be measured by mortality, long-standing illness, depression, nurse-rated health, visceral adiposity, depression, obesity, and so on [4,49,50,51]. In this study, we used the question ‘How do you feel about your current state of physical health to measure?’ The answers were divided into five categories: very unhealthy, relatively unhealthy, average, relatively healthy, and very healthy. We converted the answer options into health scores, wherein the better the health status, the higher the score.

A set of demographic and family attributes was treated as control variables. The demographic attributes include age as a continuous variable and we discarded subjects aged less than 60 years to meet the needs of the study, gender as a dichotomous variable (male = 1 and female = 0), ethnicity as a dichotomous variable (Han nationality = 1 and otherwise = 0), hukou as a dichotomous variable (city = 1 and rural = 0), and education as a dichotomous variable (educated = 1 and illiteracy = 0). Individuals’ total income in the last year was taken as logarithm and considered a continuous variable. Subjective status encompasses the individual’s family resources (Singh-Manoux, 2003). The family attribute variables include marriage as a dichotomous variable (in marriage = 1 and otherwise = 0) and number of children as a continuous variable. We took the natural logarithm of household income as continuous variable. Family economic status was considered dichotomous (average and above = 1 and otherwise = 0). Table 2 indicates the descriptive statistics of all variables.

This study first verifies the impact of subjective socioeconomic status on the health of older people, and the model is set up as follows:

(1) $$Health=\partial +\beta_{1}\;status+\beta_{2}X+\varepsilon$$

In Equation (1), health is the outcome variable, representing the physical health score. status is the core explanatory variable, representing subjective socioeconomic status. *X* is a set of control variables, and the estimated coefficient *β*_1_ is the coefficient of the effect of subjective socioeconomic status on the health of older people, based on whether it is significant or not to determine whether there are health differences between older people of different socioeconomic statuses. The positivity or negativity of *β*_1_ determines the specific manifestation of health disparities among older people of different subjective socioeconomic statuses. This is a random disturbance term.

The second model was employed to examine the health disparities among older people with different class mobility. We also use the model of Ordinary Least Squares. In this analysis, class mobility is defined as the independent variable and health score the dependent variable. Then, we explore the role played by class mobility on the impact of socioeconomic status on health in two ways: one is testing the moderating effect of class mobility using a moderating effects model; the other is dividing older people into different groups according to their mobility trajectory to make a comparison. Therefore, the health disparities among older people with different subjective socioeconomic statuses in various class mobility groups can be tested.

## 3. Results

### 3.1. Subjective Socioeconomic Status and Health Disparities

Table 3 shows that subjective socioeconomic status has a significant influence on older people’s health. In model (1), no control variables were input. This indicates that subjective socioeconomic status has a positive significant relationship with older people’s health. We first input the demographic attributes in model (2). The result shows the coefficient of subjective socioeconomic status and health is 0.231 and it remains significant at the 1% level. Then, model (3) controlled all the variables including character attributes and family attributes. The persistence of these patterns represents a sign of the robustness of the relationships between subjective socioeconomic status and health. Subjective socioeconomic status continues to show a significantly positive influence on older people’s health and the coefficient is 0.159, which means the health score of older people increases by 0.159 points for each increased level of subjective socioeconomic status. Therefore, elder people will have poorer health when possessing a lower subjective socioeconomic status.

In all the control variables, age, gender, hukou, education, income, children, household income, and family economic status have significant impacts on the health of older people. As people age, their health deteriorates. This may be related to the declining physical functions of older people. Older men have significantly better physical health than older women. Older people with non-agricultural hukou are healthier than those with agricultural hukou. Illiterate older people are in poorer physical health than educated older people. Older people have better physical health with more income. The higher the number of children, the worse the health of older people. The greater the household income, the better the health level of older people. Older people with high household economic status are healthier than others. There are only two control variables that do not have a significant influence on older people’s health: one is ethnicity, and the other is marriage. Therefore, there are no significant health disparities between older people of different ethnic groups and marital status.

In order to verify the reliability of the baseline regression results, we used a variety of methods for robustness testing. First, we replaced the analytical model with ordered-logit and ordered-probit models. These methods lead to results that are stable at the baseline regression. They show that subjective socioeconomic status has a significant positive influence on older people’s health (see model (1) and model (2) in Table 4). Older people with higher subjective socioeconomic status have significantly higher levels of health. Second, we replaced the independent variables. We introduced a new question to measure subjective socioeconomic status. The question, ‘On balance, where do you personally stand in society at the moment?’ is used to represent the subjective socioeconomic status with ten scores from 1–10 to choose from. Higher scores indicate a higher subjective socioeconomic status. A significant positive effect of subjective status on older people’s health was again verified (see model (3) in Table 4). Third, the dependent variable was substituted with a new one, which is measured in an objective way. The question is ‘In the past four weeks, how often did health problems affect your work or other daily activities’. There are five options, including ‘Always, often, sometimes, rarely and never’. We assigned health scores to the options. A lower frequency means a higher health score. This reveals that there is a significant increase in the health score as older people’s subjective socioeconomic status improves (see model (4) Table 4). Hence, all the models in Table 4 present similar results. We can confirm that subjective socioeconomic status affects older people’s health positively.

### 3.2. The Role of Class Mobility

The extent and nature of health disparities changes over the course of life [49]. From a life course perspective, a person’s subjective socioeconomic status changes with the stage of life. The linking of different periods of subjective socioeconomic status constitutes a trajectory of class mobility. The current socioeconomic status of older people may have been influenced by previous class mobility. According to the questionnaire, we can acquire class mobility trajectories in different periods. One is from the age of 14 to the present, and the other is from ten years ago to the present. Three kinds of mobility trajectories are grouped: upward, constant, and downward. Table 5 presents a clear picture of the characteristics of class mobility in the pre-elderly period. From the age of 14 to their present age, the proportion of older people who have experienced upward class mobility is 63.54%. Among all the older people in the sample, 25.86% remained in the same class and 10.60% experienced downward class mobility. From ten years ago to the present, the proportions of older people moving upwards, unchanged, and moving downwards are 47.28%, 39.64% and 13.09%, respectively. Overall, there are more older people experiencing class mobility, especially a rise in class. The shorter the time period, the more people whose status remains the same.

Are there any health disparities among older people with different class mobility? The results indicate that there are. In the period from the age of 14 to the present, older people in a constant class have a significantly lower health score than older people experiencing upward class mobility by 0.125, and those moving down the socioeconomic ladder also have obvious differences in health with respect to upward mobility. The health score gap is 0.167 (see model (1) in Table 6). Limiting the class mobility trajectory to the period from 10 years ago to the present, we acquire similar results: older people who experienced upward class mobility have a better health status than those who remained in the same class and experienced downward class mobility. Therefore, experiencing an increase in class contributes to improved health.

Different subjective socioeconomic statuses and various class mobility trajectories contribute to the health disparities of older people. After centralizing the variables of subjective socioeconomic status and the class mobility, the interaction term was created by multiplying those two centered variables. When the model includes subjective socioeconomic status, class mobility, and their interaction, we find that class mobility plays a moderating role. In the period from the age of 14 to the present, the interaction of subjective socioeconomic status and class mobility significantly affects the health of older people, wherein the coefficient is 0.072. Meanwhile, after we put class mobility and its interaction with subjective socioeconomic status in the model, the health impact factor of subjective socioeconomic status is reduced from 0.159 to 0.147 (see model (3) in Table 6). Hence, class mobility can be regarded as a moderator. In the period from ten years ago to the present, the interaction of subjective socioeconomic status and class mobility also have a significant influence on older people’s health with an impact factor of 0.088. The coefficient of influence regarding subjective socioeconomic status towards health is 0.151, which is less than 0.159. This result also supports the conclusion that class mobility moderates the health effects of subjective socioeconomic status (see model (4) in Table 6). We conclude that the impact factor of subjective socioeconomic status decreases as one’s class level increases and that class mobility helps to reduce health disparities among older people.

### 3.3. Upward Class Mobility and Health Disparities

A further point of interest relating to the prediction of health disparities concerns upward class mobility. According to the result above, we conclude that class mobility can moderate the influence of subjective socioeconomic status on health. Further analysis is provided in this article to learn which kinds of class mobility benefit the reduction in health disparities. Upward class mobility is usually considered to have a positive effect. We divided class mobility into upward mobility and others. Then, regressions in groups were adopted. In model (1) and model (2) in Table 7, the period of class mobility is from the age of 14 to the present, both the regression results indicate that subjective socioeconomic status significantly affects older people’s health, wherein the influence coefficients are significant at the level of 1%. However, the coefficient in model (1) is 0.208, which is greater than 0.110 in model (2). To ensure that the coefficients of the two models are comparable, the Chow test is adopted. We find that the difference is obvious and significant at the level of 1%. Therefore, when older people did not experience upward class mobility (model (1)), their subjective socioeconomic statuses have a greater impact on their health than those who experienced upward class mobility.

We limited the mobile class trajectory for the period from 10 years ago to the present, and the results of the different groups of tests are clearly presented in model (3) and model (4). The impact of subjective socioeconomic status on the health of older people is always significant, regardless of whether they have experienced upward mobility. The effect coefficients were significantly different in the two models and passed the Chow test, which demonstrates that the health of older people who experienced a class rise is less affected by their subjective socioeconomic status than those who did not. So, the heterogeneity test indicates that experiencing upward class mobility can play a moderating role in reducing the impact of subjective socioeconomic status on older people’s health, which in turn can contribute to the alleviation of health disparities in older people.

## 4. Discussion

We used a stepwise regression analysis to examine the effect of subjective socioeconomic status on the health of older people. The robustness tests were conducted by replacing the test model, independent, and dependent variables. The moderating effect of class mobility was tested by means of a moderating model. On this basis, the relationship between upward mobility and subjective socioeconomic status-influenced health inequalities among older people was tested. The main conclusions that we have drawn are as follows:

Firstly, health disparities exist between older people of different subjective socioeconomic statuses. The health of older people improves as their subjective socioeconomic status rises. The results passed the robustness test. This finding is consistent with existing research in China [42,52]. In addition to China, there is also extensive research worldwide, such as in developed countries such as the United States, Canada [53], and Israel [54], and in developing countries such as Mexico [55], where socioeconomic status and health are considered to be related. Thus, various subjective socioeconomic statuses contribute to health disparities in older adults in both developed and developing countries. The mechanisms of physical, lifestyle, psychosocial, and community environments have contributed to the impacts [56]. The specific indicators include access to health services [57], lifestyle [58], pressure [59], and so on.

Secondly, different class mobility trajectories lead to the creation of health disparities in older people. Those who experience upward mobility are significantly healthier than those who do not experience class mobility and those who experience downward mobility. This finding complements existing research. Additionally, the result that class mobility can somewhat mitigate the effect of subjective socioeconomic status on health indicates the robustness of the previous findings [9,60,61]. The main reason for this impact is that people’s life chances change with various socioeconomic statuses [9].

Thirdly, upward class mobility reduced the impact of subjective status on health. For older people who did not experience upward class mobility from the age of 14 to the present, their health score increases by 0.208 for each tier of subjective socioeconomic status. For older people who experienced upward mobility, their health score increases by 0.110 with subjective socioeconomic status improvement. Such results also apply to the effect of subjective socioeconomic status on the health of older people under different mobility trajectories from 10 years ago to the present. In conclusion, upward class mobility helps reduce health inequalities. People who experienced upward mobility will be in a more favorable position with respect to their health [62]. Similar results were obtained in studies on childhood socioeconomic position (SEP), where upward mobility partially compensates for disadvantages early in life [63], with education playing a larger role [52].

Although we have adopted various robustness tests to increase the reliability of the results, there are some caveats to the conclusions drawn in this study. The China General Social Survey (CGSS) data are cross-sectional, leading to some risk of endogeneity in causal inference. We will use the panel data for further validation in the future. However, this does not overshadow the strengths of this article; compared to previous literature, the main contribution of this article is the introduction of class mobility, which has been found to have a significant effect on the health of older people and to moderate the effect of subjective socioeconomic status on health. The exploration of the factors influencing the health disparities of older people combines current and early experiences, thus providing a more comprehensive analytical perspective that has set the foundation for future research.

## 5. Conclusions

China has experienced dramatically expanding inequality for over three decades since its market reforms [64]. Welfare measures helped to reduce economic, housing, and access-to-care disparities, but they were unable to completely eradicate health disparities [7]. The results of this article have wider implications for the mitigation of health disparities. Most of the literature fails to capture the deeper structural relationships among subjective socioeconomic status, class mobility, and health disparities that must be explored if we are to gain greater insight into health stratification in the social hierarchy and formulate a more effective policy response.

Health is a fundamental human right, and health disparities have become a new perspective for social stratification [9]. The existence of health disparities is detrimental to the harmonious development of society. This article demonstrates the social choice theory of the formation of health disparities in terms of both horizontal static subjective socioeconomic status and vertical dynamic class mobility. Reducing health inequalities among older people through public policy improvements is necessary. We organized various measurements of the promotion of health equality for older people along two dimensions. One dimension entails closing the gap in health resources for older people horizontally. That is, health resources should be distributed from uneven to balanced. Subjective socioeconomic status is the perception of people’s social class that represents objective socioeconomic status (such as education, occupation, and income) to a certain extent. Therefore, improving educational opportunities, income distribution, health-related behaviors, or access to health care may help to reduce health disparities [7]. All the resources relative to health should be distributed in an undifferentiated manner. The second dimension entails mitigating health disparities in older people from a longitudinal life course perspective by creating more opportunities for upward mobility for all. Class mobility is an important means of promoting social equality. Health is both a result of social stratification and a driving factor for stratification and mobility [65]. Upward class mobility decreased the impact of subjective socioeconomic status on older people’s health. Upward social mobility means that people have a higher socioeconomic status, which entails better life chances, increased access to health services, and less exposure to health risks. Conversely, the opposite is true [42]. People will have more opportunities to close this gap in health when they become old if they have experienced upward mobility, as older people’s health is influenced by the accumulation of factors from earlier stages.

## Figures and Tables

**Table 1:** Demographic characteristics of participants.

	Group	Freq.	Percent%
Gender	Male	1869	49.42
	Female	1913	50.58
Hukou	Rural	2266	59.92
	City	1516	40.08
Ethnicity	Han Nationality	3539	93.57
	Else	243	6.43
Education	Illiterate	870	23
	Else	2912	77
Marriage	In marriage	2743	72.53
	Else	1039	27.47

**Table 2:** Descriptive statistics of variables.

Variables		Obs	Mean	Std. Dev.	Min	Max
Dependent variables	Health	3782	2.985	1.073	1	5
	Health1	3782	3.455	1.242	1	5
Inependent variables	Status	3782	2.164	0.898	1	5
	Class	3782	4.033	1.762	1	10
Moderator variables	Trajectory	3782	1.471	0.679	1	3
	Trajectory1	3782	1.658	0.698	1	3
Control variables	Age	3782	69.195	7.288	60	103
	Gender	3782	0.494	0.500	0	1
	Hukou	3782	0.401	0.490	0	1
	Ethnicity	3782	0.936	0.245	0	1
	Education	3782	0.770	0.421	0	1
	Log (Income)	3782	8.311	3.566	0	16.118
	Marriage	3782	0.725	0.446	0	1
	Child	3782	2.416	1.519	0	24
	Log (Household Income)	3782	9.751	2.425	0	16.118
	Family Status	3782	0.505	0.500	0	1

**Table 3:** Results of subjective socioeconomic status’s influence on health of older people.

	(1)	(2)	(3)
Variables	Health	Health	Health
Status	0.277 ***	0.231 ***	0.159 ***
	(0.019)	(0.020)	(0.023)
Age		−0.010 ***	−0.006 **
		(0.002)	(0.003)
Gender		0.178 ***	0.163 ***
		(0.035)	(0.035)
Hukou		0.233 ***	0.084 **
		(0.037)	(0.040)
Ethnicity		0.061	0.057
		(0.071)	(0.071)
Education		0.154 ***	0.082 *
		(0.045)	(0.045)
Log (Income)			0.018 ***
			(0.006)
Marriage			−0.007
			(0.040)
Child			−0.042 ***
			(0.013)
Log (Household Income)			0.038 ***
			(0.009)
Familiy Status			0.171 ***
			(0.042)
Constant	2.387 ***	2.841 ***	2.320 ***
	(0.045)	(0.182)	(0.209)
Observations	3782	3782	3782
R-squared	0.054	0.085	0.107

Robust standard errors in parentheses; *** *p* < 0.01, ** *p* < 0.05, and * *p* < 0.1.

**Table 4:** Robustness tests.

	(1)	(2)	(3)	(4)
Variables	Health	Health	Health	Physical Health
Status	0.163 ***	0.287 ***		0.141 ***
	(0.024)	(0.043)		(0.027)
Age	−0.006 **	−0.011 **	−0.006 **	−0.016 ***
	(0.003)	(0.005)	(0.003)	(0.003)
Gender	0.169 ***	0.295 ***	0.163 ***	0.137 ***
	(0.036)	(0.062)	(0.035)	(0.040)
Hukou	0.080 *	0.140 *	0.091 **	0.211 ***
	(0.042)	(0.072)	(0.040)	(0.047)
Ethnicity	0.062	0.100	0.055	0.140 *
	(0.074)	(0.129)	(0.070)	(0.080)
Education	0.086 *	0.135 *	0.082 *	0.174 ***
	(0.047)	(0.082)	(0.045)	(0.050)
Log (Income)	0.018 ***	0.036 ***	0.018 ***	0.025 ***
	(0.006)	(0.011)	(0.006)	(0.007)
Marriage	−0.005	−0.023	−0.009	−0.005
	(0.042)	(0.072)	(0.040)	(0.046)
Child	−0.043 ***	−0.076 ***	−0.044 ***	−0.058 ***
	(0.014)	(0.024)	(0.013)	(0.015)
Log (Household income)	0.041 ***	0.070 ***	0.037 ***	0.050 ***
	(0.010)	(0.017)	(0.009)	(0.010)
Family status	0.176 ***	0.285 ***	0.185 ***	0.208 ***
	(0.043)	(0.075)	(0.039)	(0.047)
Status2			0.089 ***	
			(0.011)	
Constant			2.342 ***	3.162 ***
			(0.208)	(0.243)
Observations	3782	3782	3782	3782
R-squared			0.112	0.143

Robust standard errors in parentheses; *** *p* < 0.01, ** *p* < 0.05, and * *p* < 0.1.

**Table 5:** Class mobility of older people.

Period	Trajectory	Freq.	Percent%	Cum.%
From the age of 14 to the present	Upward	2403	63.54	63.54
	Constant	978	25.86	89.40
	Downward	401	10.60	100.00
From 10 years ago to the present	Upward	1788	47.28	47.28
	Constant	1499	39.64	86.91
	Downward	495	13.09	100.00

**Table 6:** Test of role of class mobility.

	(1)	(2)	(3)	(4)
Variables	Health	Health	Health	Health
Unchanged1	−0.125 ***		−0.074 *	
	(0.039)		(0.040)	
Downward1	−0.167 ***		−0.104 *	
	(0.056)		(0.056)	
Unchanged2		−0.085 **		−0.059
		(0.036)		(0.036)
Downward2		−0.258 ***		−0.216 ***
		(0.054)		(0.054)
Status			0.147 ***	0.151 ***
			(0.024)	(0.024)
Interaction1			0.072 ***	
			(0.028)	
Interaction2				0.088 ***
				(0.027)
Control variables	Yes	Yes	Yes	Yes
Constant	2.535 ***	2.460 ***	2.398 ***	2.373 ***
	(0.213)	(0.211)	(0.211)	(0.210)
Observations	3782	3782	3782	3782
R-squared	0.099	0.102	0.110	0.114

Robust standard errors in parentheses; *** *p* < 0.01, ** *p* < 0.05, and * *p* < 0.1.

**Table 7:** Heterogeneity test.

	(1)	(2)	(3)	(4)
Variables	Health	Health	Health	Health
Status	0.208 ***	0.110 ***	0.170 ***	0.120 ***
	(0.041)	(0.029)	(0.032)	(0.035)
Age	−0.003	−0.008 **	−0.007 *	−0.003
	(0.005)	(0.003)	(0.004)	(0.004)
Gender	0.144 **	0.183 ***	0.209 ***	0.117 **
	(0.057)	(0.044)	(0.046)	(0.052)
Hukou	0.079	0.103 **	0.106 *	0.079
	(0.069)	(0.051)	(0.056)	(0.060)
Ethnicity	−0.003	0.102	−0.043	0.168 *
	(0.124)	(0.086)	(0.098)	(0.101)
Education	0.256 ***	−0.007	0.182 ***	−0.000
	(0.077)	(0.056)	(0.066)	(0.062)
Log (Income)	0.027 ***	0.013 *	0.028 ***	0.010
	(0.010)	(0.008)	(0.009)	(0.009)
Marriage	−0.010	−0.014	−0.038	0.030
	(0.066)	(0.051)	(0.054)	(0.060)
Children	−0.044 *	−0.041 ***	−0.049 ***	−0.041 **
	(0.024)	(0.014)	(0.019)	(0.018)
Log (Household income)	0.016	0.050 ***	0.017	0.055 ***
	(0.016)	(0.011)	(0.013)	(0.012)
Family status	0.156 **	0.173***	0.217 ***	0.116*
	(0.069)	(0.052)	(0.057)	(0.061)
Constant	2.032 ***	2.548 ***	2.436 ***	2.153 ***
	(0.342)	(0.266)	(0.279)	(0.319)
Observations	1379	2403	1994	1788
R-squared	0.142	0.089	0.150	0.075
Chow test	2.22 ***		2.84 ***	

Robust standard errors in parentheses; *** *p* < 0.01, ** *p* < 0.05, and * *p* < 0.1.

## Data Availability

The datasets generated and/or analyzed during the current study are available in the CGSS repository, http://cgss.ruc.edu.cn/index.htm (accessed on 9 May 2021).
